# Loss of PML nuclear bodies in familial amyotrophic lateral sclerosis-frontotemporal dementia

**DOI:** 10.1038/s41420-023-01547-2

**Published:** 2023-07-15

**Authors:** Francesco Antoniani, Marco Cimino, Laura Mediani, Jonathan Vinet, Enza M. Verde, Valentina Secco, Alfred Yamoah, Priyanka Tripathi, Eleonora Aronica, Maria E. Cicardi, Davide Trotti, Jared Sterneckert, Anand Goswami, Serena Carra

**Affiliations:** 1 Department of Biomedical, Metabolic and Neural Sciences, University of Modena and Reggio Emilia, Modena, Italy; 2 Centro Interdipartimentale Grandi Strumenti (CIGS), University of Modena and Reggio Emilia, Modena, Italy; 3 Institute of Neuropathology, RWTH Aachen University Hospital, Aachen, Germany; 4 Amsterdam UMC location University of Amsterdam, Department of (Neuro)Pathology, Amsterdam Neuroscience, Meibergdreef 9, Amsterdam, the Netherlands; 5 Weinberg ALS Center, Vickie and Jack Farber Institute for Neuroscience, Department of Neuroscience, Thomas Jefferson University, Philadelphia, PA, USA; 6 Center for Regenerative Therapies TU Dresden, Technische Universität Dresden, Dresden, Germany; 7 Medical Faculty Carl Gustav Carus of TU Dresden, Dresden, Germany; 8 Department of Neurology, Center for Motor Neuron Biology and Disease, Columbia University, 10032 New York, NY USA; 9 Department of Neurology, Eleanor and Lou Gehrig ALS Center, Columbia University, 10032 New York, NY USA

**Keywords:** Mechanisms of disease, Molecular biology

## Abstract

Amyotrophic Lateral Sclerosis (ALS) and Frontotemporal Dementia (FTD) are two neurodegenerative disorders that share genetic causes and pathogenic mechanisms. The critical genetic players of ALS and FTD are the TARDBP, FUS and C9orf72 genes, whose protein products, TDP-43, FUS and the C9orf72-dipeptide repeat proteins, accumulate in form of cytoplasmic inclusions. The majority of the studies focus on the understanding of how cells control TDP-43 and FUS aggregation in the cytoplasm, overlooking how dysfunctions occurring at the nuclear level may influence the maintenance of protein solubility outside of the nucleus. However, protein quality control (PQC) systems that maintain protein homeostasis comprise a cytoplasmic and a nuclear arm that are interconnected and share key players. It is thus conceivable that impairment of the nuclear arm of the PQC may have a negative impact on the cytoplasmic arm of the PQC, contributing to the formation of the cytoplasmic pathological inclusions. Here we focused on two stress-inducible condensates that act as transient deposition sites for misfolding-prone proteins: Promyelocytic leukemia protein (PML) nuclear bodies (PML-NBs) and cytoplasmic stress granules (SGs). Upon stress, PML-NBs compartmentalize misfolded proteins, including defective ribosomal products (DRiPs), and recruit chaperones and proteasomes to promote their nuclear clearance. SGs transiently sequester aggregation-prone RNA-binding proteins linked to ALS-FTD and mRNAs to attenuate their translation. We report that PML assembly is impaired in the human brain and spinal cord of familial C9orf72 and FUS ALS-FTD cases. We also show that defective PML-NB assembly impairs the compartmentalization of DRiPs in the nucleus, leading to their accumulation inside cytoplasmic SGs, negatively influencing SG dynamics. Although it is currently unclear what causes the decrease of PML-NBs in ALS-FTD, our data highlight the existence of a cross-talk between the cytoplasmic and nuclear PQC systems, whose alteration can contribute to SG accumulation and cytoplasmic protein aggregation in ALS-FTD.

## Introduction

Amyotrophic Lateral Sclerosis (ALS) is a neurodegenerative disorder caused by interactions between genetic factors and external environmental factors. So far, more than 20 genes have been associated with ALS [1]. The hexanucleotide GGGGCC repeat expansion in the *C9orf72* gene is the most common mutation, responsible for 30–50 % of familial ALS cases. The mutation of the *C9orf72* gene is also frequently associated with Frontotemporal Dementia (FTD), which unexpectedly shows a strong molecular overlap with ALS. Besides *C9orf72*, mutations in the genes coding for the RNA-binding proteins (RBPs) TDP-43 (TAR DNA‐binding protein 43) and FUS (fused in sarcoma) have been found in circa 15% of all patients [1, 2]. Thus, ALS and FTD are currently considered as the two extremes of an overlapping spectrum disorder called ALS-FTD, characterized by the progressive loss of upper and lower motor neurons (MNs), as well as by the selective degeneration of neurons in frontal and temporal lobes [3].

ALS and FTD are characterized by the accumulation of aggregated proteins that can exert toxic effects [4]. The pathological protein inclusions mainly contain TDP-43, FUS, and ubiquitinated proteins [5]. TDP-43 pathology is observed also in ALS and FTD patients with *C9orf72* mutation, reflecting the pathological overlap between these two diseases [6]. Although the origin of TDP-43 and FUS pathological inclusions is still debated, their protein aggregation is considered a key pathogenic event in ALS and FTD, along with impairment of RNA metabolism, disruption of the nuclear transport and defective repair of DNA lesions [1].

TDP-43 and FUS are nuclear RNA-binding proteins (RBPs); however, their aggregation mainly takes place in the cytoplasm. Thus, most of the studies focus on the understanding of how cells control TDP-43 and FUS aggregation in the cytoplasm, overlooking how dysfunctions occurring at the nuclear level may influence the maintenance of protein solubility outside of the nucleus. Yet, protein quality control (PQC) systems that recognize and deal with aggregation-prone proteins simultaneously operate in the cytoplasm and nucleus and share key players, including chaperones of the heat shock protein families and proteasomes [7–9]. Thus, it is conceivable that impairment of the nuclear arm of the PQC may have a negative impact on the cytoplasmic arm of the PQC, contributing to the formation of the cytoplasmic pathological inclusions.

Upon stress conditions that promote protein misfolding, mammalian cells transiently store aggregation-prone proteins in specific deposition sites that are formed both in the cytoplasm and nucleus [10, 11]. Two examples of transient deposition sites for misfolding-prone proteins that are induced upon stress include Promyelocytic leukemia protein (PML) nuclear bodies (PML-NBs) in the nucleus [12, 13] and stress granules (SGs) in the cytoplasm [14].

PML-NBs are an archetype for nuclear condensates that form through phase separation mechanisms and appear as spheres of circa 0.1 μm in diameter within the nucleoplasm [15]. The main component of PML-NBs is the PML protein, which is conjugated to SUMO1 and contains a SUMO interacting motif. SUMO/SIM interactions provide the molecular glue to build up PML-NBs and stabilize interactions with other components [15]. Besides regulating chromatin dynamics, transcription and genome maintenance [15], PML-NBs also promote the clearance of misfolded and disease-causing mutated proteins and have been suggested to play a protective role in neurodegenerative diseases [16]. SGs are cytoplasmic RNA-protein condensates that form in response to stress and transiently store stalled pre-initiation complexes, signaling molecules, nuclear proteins, including aggregation-prone TDP-43 and FUS, indirectly affecting mRNA translation, cellular metabolism and survival [17, 18]. SGs are highly dynamic and rapidly disassemble upon stress relief, enabling the restoration of protein synthesis and RNA metabolism [17, 18].

Although apparently unrelated, cytoplasmic SGs and PML-NBs are interconnected via the so-called SUMO-targeted ubiquitin ligase (STUbL) pathway, in which the two post-translational modifications SUMOylation and ubiquitylation, and the RNF4 E3 ligase, cooperate to clear damaged proteins [19]. RNF4 interacts with the SUMO-primed substrates and PML [12, 13]. Upon stress several aggregation-prone RBPs that are recruited inside SGs, including TDP-43 and FUS, undergo SUMO-primed, RNF4-dependent ubiquitylation and the fraction of SUMO-primed RBPs is targeted to PML-NBs for proteasome-mediated degradation [20]. Inhibition of SUMOylation and depletion of either RNF4 and PML impairs the nuclear clearance of SUMO-primed RBPs, favoring their accumulation inside SGs and delaying SG disassembly [20]. Based on these findings, it has been proposed that compartmentalization of SUMO-primed aggregation-prone proteins at PML-NBs avoids their accumulation and potential aggregation within SGs, maintaining their dynamics. Of note, the accumulation of SGs with delayed disassembly kinetics (referred to as aberrant SGs) has been repeatedly reported in ALS and FTD cell models and has been suggested to contribute to the formation of the pathological aggregates observed in the patients [21–24], although protein aggregation can also occur in the cytoplasm independently of SGs [25, 26]. Yet, whether changes at the levels of PML occur in ALS-FTD is unknown.

Here, we report that PML assembly is impaired in the human brain and spinal cord of familial *C9orf72* and FUS ALS-FTD cases. To investigate whether/how alterations of PML-NBs may affect PQC and SG dynamics in the cytoplasm, we used different cell models, including iPSC-derived motor neurons expressing expanded *C9orf72*. We demonstrate that dysfunction of the nuclear PQC due to PML depletion promotes the accumulation of misfolded proteins inside SGs, impairing their disassembly. Although it is currently unclear what causes the decrease of PML-NBs in ALS-FTD, our data highlight the existence of a cross-talk between the cytoplasmic and nuclear PQC systems, whose alteration can contribute to SG accumulation and cytoplasmic protein aggregation in ALS-FTD.

## Results

### Impairment of PML-NBs in familial *C9orf72* and FUS ALS-FTD

Previous studies identified a correlation between decreased expression of PML and SUMO, which is required for PML assembly into nuclear condensates, impaired SG dynamics, and accumulation of ALS-linked proteins, including *C9orf72* dipeptide repeats, TDP-43 and mutated FUS [20, 27]. Yet, it is currently unknown whether alterations at the level of PML-NBs occur in familial ALS-FTD, which may further contribute to disease progression. Given that PML-NBs were suggested to participate in the clearance of TDP-43 and FUS [20], we selected for our study brain and spinal cord samples from familial *C9orf72* ALS-FTD cases, which are also characterized by TDP-43 pathology [6, 28], and FUS ALS-FTD cases, where FUS pathology was dominant, to explore whether changes at the levels of PML-NBs occurred.

**Fig. 1:**
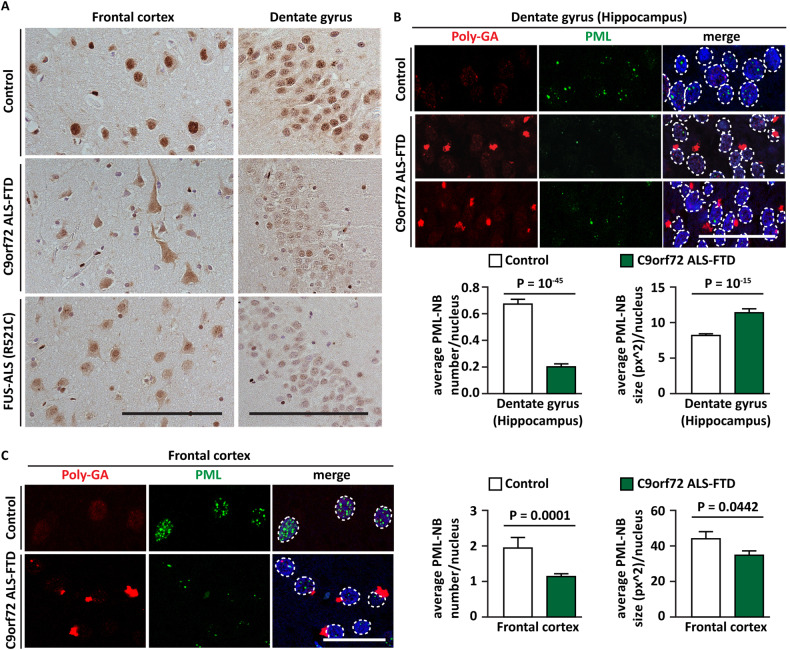
The number of PML-NBs is reduced in the frontal cortex and hippocampus of C9orf72 ALS-FTD patients. **A** DAB immunolabelling using PML antibody (paraffin sections) on the frontal cortex and dentate gyrus (hippocampus) of a representative control section (upper panel), showing strong nuclear immunoreactivity of PML, of C9orf72 ALS-FTD (middle panel) and of FUS-ALS-FTD (lower panel), showing an overall reduced nuclear immunoreactivity. *n* = 3 control, *n* = 4 C9orf72-ALS-FTD, *n* = 3 FUS-ALS-FTD. Scale bar is 50 μm. Co-immunolabeling using poly-GA and PML antibodies showing a significant decrease of PML-NB number in hippocampal dentate gyrus (**B**) and cortical (**C**) neurons harboring polyGA aggregates in C9orf72 ALS-FTD brain, compared to the controls (upper panel). *n* = 3 control, *n* = 4 C9orf72-ALS-FTD, *n* = 3 FUS-ALS-FTD. Scale bar is 20 μm. PML and nuclei were segmented using ScanR (Olympus) and the number and size of PML-NBs per nucleus was automatically quantified. Quantification of PML-NB average number and size (px^2) is shown. Number of cells analyzed: dentate gyrus control (1263); dentate gyrus C9orf72 ALS-FTD (1523) (**B**); frontal cortex control (122); frontal cortex C9orf72 ALS-FTD (682) (**C**).

First, we analyzed the immunolabelling patterns and the number of PML-NBs in frontal cortex and hippocampal postmortem tissue obtained from a well-characterized cohort of familial *C9orf72* ALS-FTD and FUS-ALS-FTD patients (Supplementary Table 1). DAB immunohistochemistry performed using a specific PML antibody revealed a strong nuclear immunoreactivity in the cortical pyramidal and hippocampal dentate gyrus neurons of the control patient’s brain. The PML nuclear immunoreactivity was largely reduced in the pyramidal and hippocampal dentate gyrus neurons of both the *C9orf72*- and FUS-ALS-FTD patients’ brain (Fig. 1A).

Next, to test whether the decreased levels of PML nuclear immunoreactivity observed was associated with expanded G_4_C_2_ repeats in *C9orf72*-ALS-FTD, we used double immunofluorescence labeling with established polyGA and PML antibodies. Consistent with the DAB IHC, co-immunolabelling analysis using both the antibodies followed by automatic segmentation and quantifications revealed a significant decrease of PML-NB number in both hippocampal dentate gyrus and cortical neurons in C9orf72 ALS-FTD brain compared to the controls (Fig. 1B, C).

**Fig. 2:**
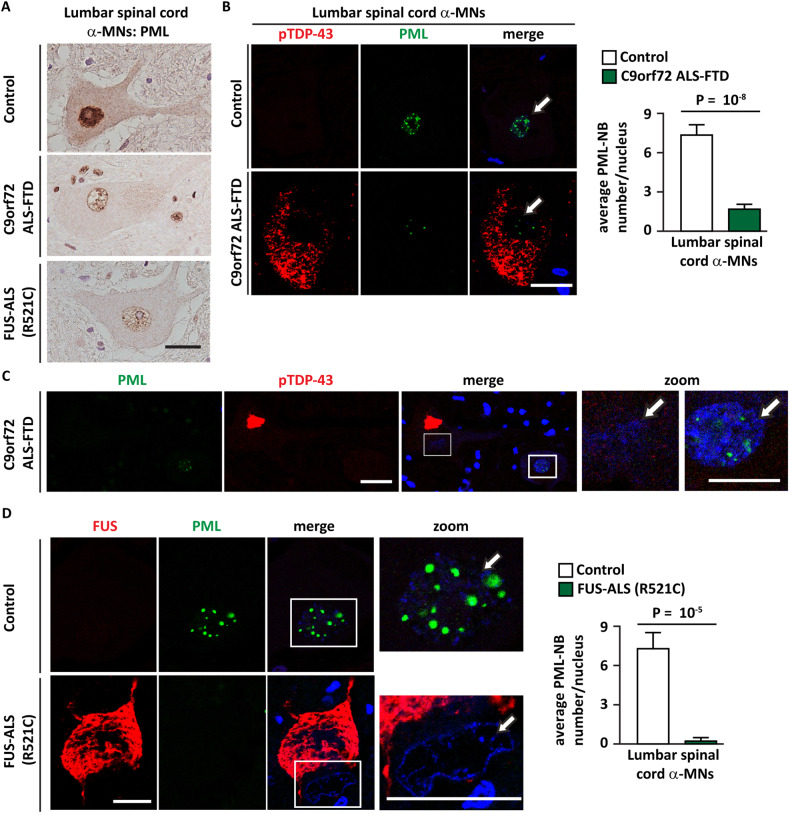
The number of PML-NBs is reduced in lumbar spinal cord α-MNs of C9orf72 ALS-FTD and FUS ALS-FTD (R521C). **A** DAB immunolabelling using PML antibody (paraffin sections) on the lumbar spinal cord. α-MNs of a representative control section (upper panel) show strong nuclear immunoreactivity of PML, whereas α-MNs of C9orf72 ALS-FTD (middle panel) and of FUS-ALS-FTD (lower panel) show an overall drastically reduced nuclear immunoreactivity. *n* = 3 control, *n* = 4 C9orf72-ALS-FTD, *n* = 3 FUS-ALS-FTD. Scale bar is 20 μm. **B**, **C** Co-immunolabeling using phosphorylated TDP-43 (pTDP-43) and PML antibodies in the lumbar spinal cord α-MNs of a representative control section (upper panel) and C9orf72 ALS-FTD (lower panel). The white arrows point to nuclear PML immunoreactivity (**B**). PML-NBs were automatically segmented and quantified. **B** The average number of PML-NBs/nucleus is shown. Number of lumbar spinal cord a-MNs analyzed: control (24); C9orf72 ALS-FTD (45); staining p-TDP43 and PML. **C** Note the strongly reduced immunolabelling of PML-NBs in the α-MNs harboring pTDP-43 aggregates compared to the adjacent α-MNs without any pTDP-43 aggregates. Serial paraffin sections; scale bar is 40 μm (zoom:20 μm). *n* = 3 control, *n* = 4 C9orf72-ALS-FTD. **D** Co-immunolabeling using FUS and PML antibodies in the lumbar spinal cord α-MNs. The white arrow shows lack of nuclear PML immunoreactivity in the α-MNs harboring FUS aggregates. The average number of PML-NBs/nucleus is shown. Serial paraffin sections; scale bar is 30 μm (zoom:15 μm). Number of lumbar spinal cord a-MNs analyzed: control (12); FUS-ALS (R521C) (15); staining FUS and PML.

Then, we analyzed PML immunoreactivity and we quantified PML-NB number in human lumbar spinal cord alfa (α) motor neurons (α-MNs) obtained from normal controls, as well as from the familial *C9orf72* ALS-FTD or FUS R521C-ALS patients. Although we were not able to detect polyGA aggregates in the surviving α-MNs from the lumbar spinal cord of these *C9orf72*-ALS-FTD cases, we noticed that such α-MNs were dominated with characteristic phosphorylated TDP-43 (pTDP-43) pathology. Thus, we speculated a similar profound alteration of PML-NBs in these α-MNs. As expected, and confirming the observations in pyramidal and dentate gyrus neurons, DAB IHC revealed a strongly reduced PML nuclear immunoreactivity in the lumbar spinal cord α-MNs of both *C9orf72* and FUS-ALS-FTD patients compared to the controls (Fig. 2A). Furthermore, consistent with the DAB IHC (Fig. 2A), co-immunolabelling of PML antibody together with either the pTDP-43 antibody in *C9orf72* (Fig. 2B, C) or with FUS antibody in FUS-ALS-FTD cases (Fig. 2D) revealed an overall profound reduction of PML-NB number in the α-MNs compared to the controls. Interestingly α-MNs harboring either the pTDP-43 or FUS aggregates specifically showed reduced PML immunoreactivity compared to the adjacent α-MNs devoid of any aggregates (Fig. 2C, D, arrows). These results show that PML-NB numbers inversely correlate with the presence of pathogenic aggregates in familial ALS-FTD.

### Reduced number of PML-NBs delays SG disassembly

The in vivo data show a correlation between the loss of PML-NBs and the presence of cytoplasmic aggregates. In mammalian cells, PML-NBs represent an important PQC site that sequesters mutated disease-linked and misfolded proteins to promote their clearance [12, 13]; this, in turn, opens the possibility that PML-NB loss may negatively influence the cells ability to compartmentalize and clear aggregation-prone proteins in the nucleus. Whether these aggregating species will be confined to the nucleus or may be redirected to the cytoplasm for sequestration and processing is unclear. In the context of ALS-FTD, the cytoplasmic inclusions enriched for TDP-43 have been suggested to originate with distinct mechanisms. First, a SG-independent direct deposition pathway in which the proteins mislocalize to the cytoplasm, where they oligomerize and form fibrillar aggregates. Second, a SG-dependent pathway in which the proteins are recruited into SGs that fail to disassemble and convert into an aggregated state. This hypothesis is supported by the finding that the chronic induction of SGs leads to the formation of cytosolic inclusions that contain TDP-43 and other components found in the patient inclusions and cause neurotoxicity [24].

As mentioned earlier, PML and SG dynamics are both influenced by protein SUMOylation. Depletion of PML and inhibition of SUMOylation, which is required to promote PML-NB assembly, delay SG disassembly with yet unclear mechanisms [20]. Whether this is hinting to a crosstalk between the cytoplasmic and nuclear arms of the PQC is unclear.

**Fig. 3:**
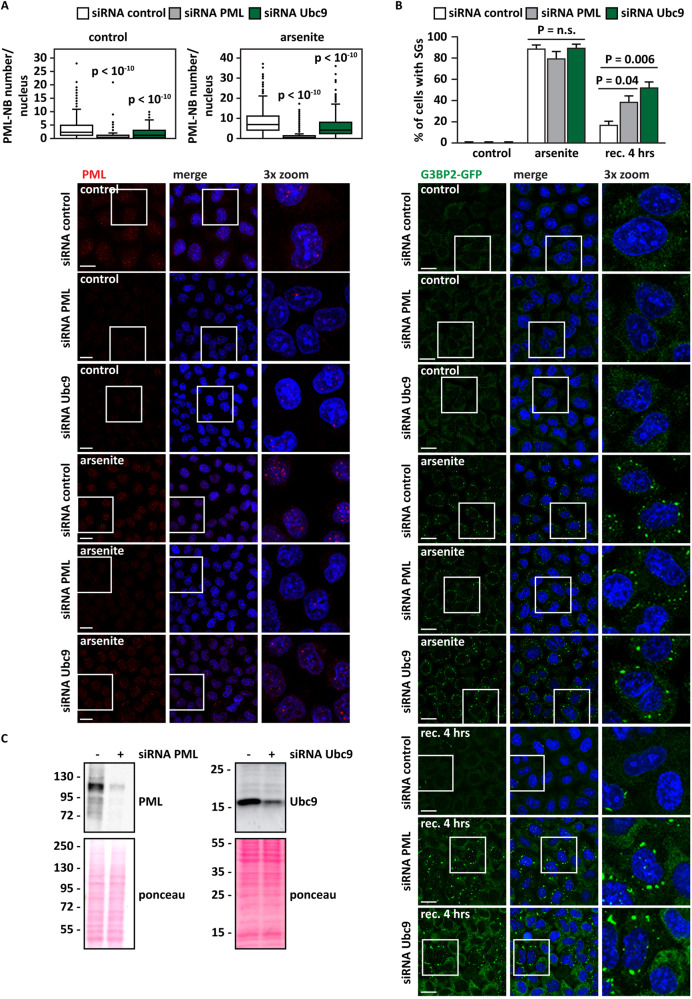
Decreased PML-NB number correlates with SG persistence in HeLa cells. **A**, **B** G3BP2-GFP HeLa Kyoto cells were lipofected with a non-targeting control siRNA (siRNA control) or with siRNA specific for PML or Ubc9, respectively. 72 h post-transfection, cells were either left untreated or treated with sodium arsenite (500 μM) for 45 min. Where indicated cells were allowed to recover in drug-free medium for 4 h (rec. 4 h). Cells were fixed (**A**, **B**), subjected to immunostaining with a PML-specific antibody (**A**). Nucleic acid was labeled using DAPI (**A**, **B**). PML-NBs were automatically segmented and quantified with ScanR (A). The number of PML-NBs analyzed is reported: siRNA control (1026, control; 1161, arsenite); siRNA PML (1042, control; 1260, arsenite); siRNA Ubc9 (1098, control; 1164, arsenite). One-way ANOVA, Post-hoc Bonferroni-Holm test. Panel B shows the percentage of SG-positive cells and representative images. *n* = 3, +/− SEM; P = n.s. (non-significant). One-way ANOVA, Post-hoc Bonferroni-Holm test. **C** Immunoblotting showing PML (left panel) and Ubc9 (right panel) siRNA efficacy in mammalian cells. Ponceau staining shows equal loading. **A**, **B** Scale bar is 10 μm.

To better understand how PML-NB dysfunction affects SG dynamics, we depleted by siRNA transfection PML and UBE2I/Ubc9, the sole E2 SUMO conjugating enzyme, in HeLa Kyoto cells stably expressing the SG marker GFP-G3BP2 [29, 30]. Depletion of PML and UBE2I/Ubc9 significantly decreased the number of PML-NBs in resting cells (Fig. 3A, control). They also significantly lowered the total number of PML-NBs in cells treated with arsenite (Fig. 3A, arsenite), a known inducer of SG and PML-NB assembly. While PML and Ubc9 depletion did not induce spontaneous SG formation, nor abrogated the ability of the cells to form SGs upon arsenite treatment, they increased the percentage of cells that failed to disassemble SGs during the stress recovery phase (Fig. 3B, C). These data are in line with previous findings obtained in HeLa cells [20] and further establish a link between loss of PML-NBs and delayed SG disassembly. Yet, whether SG accumulation is due to an impairment of the nuclear PQC function of PML is unclear.

### Failure to compartmentalize DRiPs at PML-NBs leads to their accumulation inside SGs

PML-NBs and SGs are induced upon severe stress conditions that attenuate general translation, such as treatment with sodium arsenite or MG132 (Supplementary Fig. 1A, B). These stress conditions concomitantly induce the conjugation with SUMO2/3 of many proteins (Supplementary Fig. 1A, B). Although the functional significance of protein SUMOylation upon stress is still unclear, recent evidence suggests that it promotes protein solubility and facilitates the restoration of protein homeostasis during the stress recovery phase [31].

**Fig. 4:**
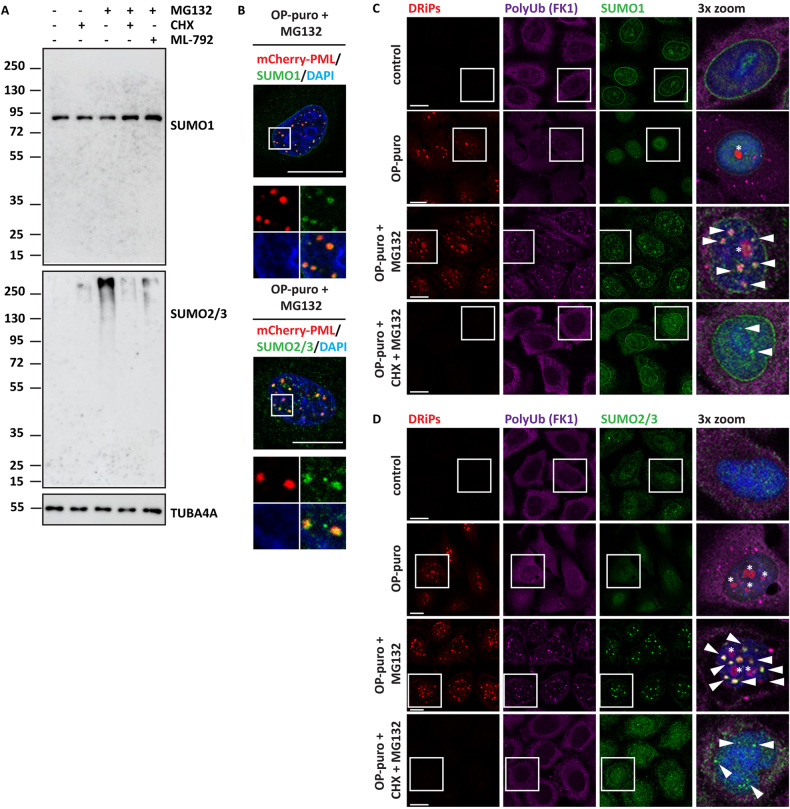
Upon proteotoxic stress conditions that induce SGs, a fraction of newly synthesized SUMOylated proteins, including DRiPs, is compartmentalized at PML-NBs. **A** HeLa Kyoto cells were either left untreated or exposed to cycloheximide (CHX; 50 μg/ml), MG132 (20 μM), ML-792 (1 μM) alone or combined for 3 h. Then, protein extracts were subjected to SDS-PAGE followed by immunoblotting with antibodies specific for SUMO1 and SUMO2/3. TUBA4A was used as loading control. **B** HeLa Kyoto cells were transfected with a DNA coding for mCherry-PML. 24 h post-transfection, cells were treated with OP-puro (25 μg/ml) and MG132 (20 μM) for 3 h. Cells were fixed and immunostained with antibodies specific for SUMO1 or SUMO2/3. Nucleic acid was labeled using DAPI. Scale bar is 10 μm. **C**, **D** HeLa Kyoto cells were either left untreated (control) or exposed to OP-puro (25 μg/ml), or OP-puro (25 μg/ml) and MG132 (20 μM) for 3 h. Where indicated, cells were co-treated with CHX (50 μg/ml) to inhibit translation. Cells were fixed and subjected to click chemistry to visualize DRiPs, followed by immunostaining with antibodies specific for polyubiquitinated proteins (FK1) and either SUMO1 (**C**) or SUMO2/3 (**D**). Scale bar is 10 μm. White arrowheads show SUMO1-positive puncta; SUMO1 is used as a proxy for PML-NBs. Asterisks show nucleoli enriched for DRiPs.

A large fraction of the proteins that undergo SUMOylation upon proteotoxic stress conditions is represented by newly synthesized proteins, including defective ribosomal products (DRiPs) [12, 32, 33]. DRiPs originate due to errors occurring during DNA replication, transcription and translation, including premature termination and amino acid misincorporation; they cannot reach their native folding state and are highly aggregation-prone [34, 35]. To differentiate between pre-existing proteins and newly synthesized proteins, we co-treated HeLa cells with MG132 and the translation inhibitor cycloheximide (CHX). Translation inhibition decreased the accumulation of SUMO2/3 conjugates compared to single treatment with MG132 (Fig. 4A). As additional control, pre-treatment of the cells with the SUMOylation inhibitor ML-792 also prevented the accumulation of SUMO2/3 conjugates upon proteasome inhibition (Fig. 4A).

The clearance of these SUMOylated proteins, including DRiPs, is thought to occur mainly at the nuclear level, in particular at PML-NBs, where these proteins are transiently stored for later clearance via the STUbl pathway [12, 32, 36]. We thus investigated the subcellular distribution of DRiPs that were labeled with the puromycin analog OP-puro [12]. Upon cell treatment with MG132 and OP-puro, we found that SUMO1 and SUMO2/3 colocalized with mCherry-PML at PML-NBs (Fig. 4B); in addition, DRiPs colocalized with SUMO1, SUMO2/3 and poly-ubiquitin chains at PML-NBs (Fig. 4C, D). As a control, the accumulation of these proteins at PML-NBs was prevented by co-incubation of the cells with MG132 and CHX (Fig. 4C, D). These colocalization data are in line with previous findings demonstrating that, upon stress, a fraction of DRiPs undergoes SUMOylation and ubiquitylation and is targeted to PML-NBs for subsequent proteasome-mediated clearance [12, 32].

**Fig. 5:**
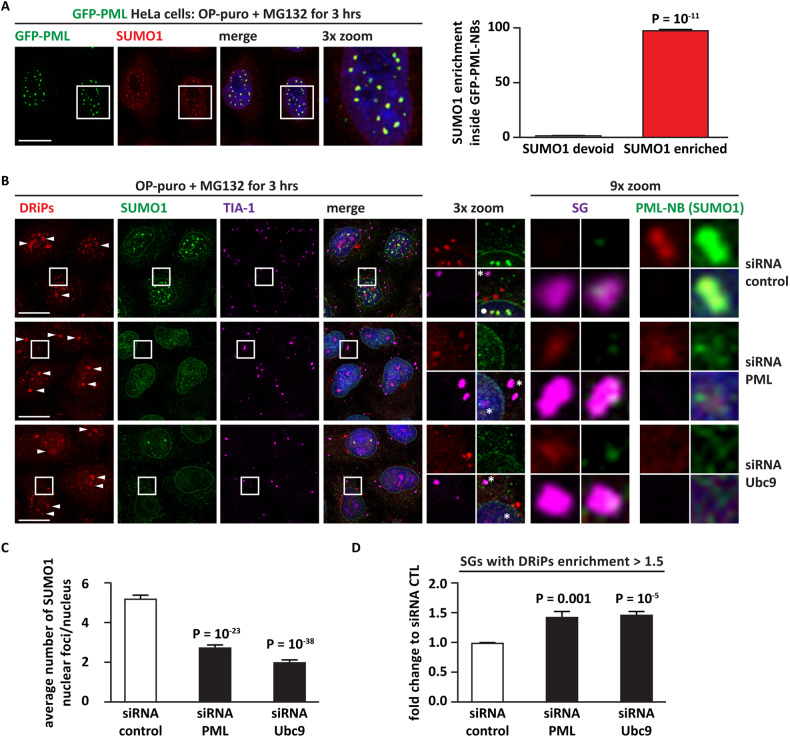
Depletion of PML or Ubc9 leads to the accumulation of DRiPs inside SGs. **A** HeLa Kyoto cells stably expressing GFP-PML were treated with OP-puro (25 μg/ml) and MG132 (20 μM) for 3 h. Cells were fixed and stained for SUMO1. Nucleic acid was labeled using DAPI. GFP-PML-NBs were automatically segmented, followed by quantification of the enrichment for SUMO1 inside each GFP-PML-NB. Enrichment < 1 corresponds to GFP-PML-NBs devoid of SUMO1; enrichment > 1 corresponds to GFP-PML-NBs colocalizing with SUMO1. The GFP-PML-NB number analyzed was as follows: 2388 (sample 1); 3636 (sample 2); 3549 (sample 3). **B** HeLa Kyoto cells were lipofected with a non-targeting siRNA control (siRNA control) or with siRNA secific for PML and Ubc9; 72 h post-transfection, cells were treated with OP-puro (25 μg/ml) and MG132 (20 μM) for 3 h. Cells were fixed and subjected to click chemistry to visualize DRiPs, followed by immunostaining with antibodies specific for the SG marker TIA1 and SUMO1. Considering that SUMO1-PML assembles into nuclear bodies, SUMO1 simultaneously enables to visualize SUMO1-conjugated proteins and SUMOylated PML-NBs. Note the three dotted-like structures positive for SUMO1 and DRiPs in the nucleus of cells transfected with siRNA CTL (white dot in the 3x zoom panel). Note also that SGs in these cells do not colocalize with DRiPs (* in the 3x zoom panel). Instead, in cells treated with siRNA PML and siRNA Ubc9, note the absence of SUMO1 and DRiPs positive puncta in the nucleus (* in the 3x zoom panel) and the colocalization of SGs with DRiPs (* in the 3x zoom panel). Nucleic acid was labeled using DAPI. White arrowheads show accumulation of DRiPs inside nucleoli, as previously published [12]. Scale bar is 10 μm. **C** Quantification of the number of SUMO1 nuclear foci in cells from A. Nuclear foci were segmented using the SUMO1 signal. Number of nuclei analyzed: 676 (siRNA control); 546 (siRNA PML); 546 (siRNA Ubc9). The average number of SUMO1 nuclear foci/nucleus is shown, +/− s.e.m. One-way ANOVA followed by Post-hoc Bonferroni-Holm test. **D** Quantification of the enrichment of DRiPs inside SGs in cells from (**A**). SGs were segmented using the TIA1 signal. Number of SGs segmented and quantified in three independent experiments: 3708 (siRNA control); 5710 (siRNA PML); 4833 (siRNA Ubc9). SGs with DRiPs’ enrichment > 1.5 is shown, +/− s.e.m. One-way ANOVA followed by Post-hoc Bonferroni-Holm test.

DRiPs include truncated proteins with low molecular weight that can freely shuttle through the nuclear pore complexes [12]. We thus asked whether destabilization of PML-NBs may favor the nuclear egression of DRiPs, promoting their aggregation in the cytoplasm. To address this question, we depleted either PML or Ubc9, which both decrease the number of PML-NBs (Fig. 3). Then, we exposed HeLa cells to MG132 and OP-puro, which cause the accumulation of DRiPs and concomitantly induce cytoplasmic SGs. After cell fixation, we stained SGs, using TIA-1 as a marker, DRiPs, and SUMO1. Since PML conjugation to SUMO1 is essential for its assembly into PML-NBs and since PML also binds to other SUMOylated proteins [37], we used the SUMO1 antibody as a proxy to visualize PML-NBs and simultaneously monitor the compartmentalization of SUMOylated proteins (including DRiPs) in the nucleus (see also Fig. 2B). First, we used HeLa Kyoto cells stably expressing GFP-PML to quantify the colocalization of SUMO1 with PML-NBs. GFP-PML HeLa Kyoto cells were treated with MG132 and OP-puro, followed by fixation and staining with a SUMO1 antibody. GFP-PML-NBs were automatically segmented and their enrichment for SUMO1 was quantified. Figure 5A shows that 98.7 % of GFP-PML-NBs colocalize with SUMO1. This enables us to use the SUMO1 antibody as a proxy for the visualization of PML-NBs under our experimental conditions. Concerning DRiPs distribution, in cells transfected with a non-targeting siRNA control and exposed to MG132 and OP-puro, we observed the formation of cytoplasmic SGs devoid of DRiPs (Fig. 5B, C, asterisk). As expected [12, 32], DRiPs accumulated inside nucleoli (Fig. 5B, arrowheads) and at SUMO1-positive PML-NBs (Fig. 5B, C). Upon PML or Ubc9 depletion, the number of SUMO1-positive nuclear foci (used as a proxy for PML-NBs) significantly decreased and SUMO1 and DRiPs accumulated in nucleoli and in the nucleoplasm, where however no clear sequestration in punctate structures was observed (Fig. 5B, C). This was paralleled by the accumulation of DRiPs inside cytoplasmic SGs upon PML or Ubc9 depletion (Fig. 5B, D). Of note, we and others previously published that the accumulation of DRiPs inside SGs changes their biochemical properties and converts SGs from dynamic RNA-protein-based condensates into protein-enriched aggregated-like structures [30, 38]. SGs enriched for DRiPs fail to disassemble and are referred to as aberrant SGs [30, 38]. Although we cannot exclude that a fraction of DRiPs might also accumulate at higher rate inside the nucleoli as a consequence of PML and Ubc9 depletion, together our data link impaired PML-NB assembly and decreased nuclear PQC with the accumulation of DRiPs inside SGs and SG accumulation.

### Cells overexpressing expanded C9orf72 G_4_C_2_ repeats accumulate SGs and display a lower number of PML-NBs

Since arsenite and MG132 cause the accumulation of aggregation-prone proteins and concomitantly the assembly of SGs and PML-NBs [15], it is difficult to assess how the nuclear PQC function of PML-NBs affects cytoplasmic SGs. To further address this question, we used a cellular model in which the formation of SGs could be induced without treatment with arsenite or MG132.

**Fig. 6:**
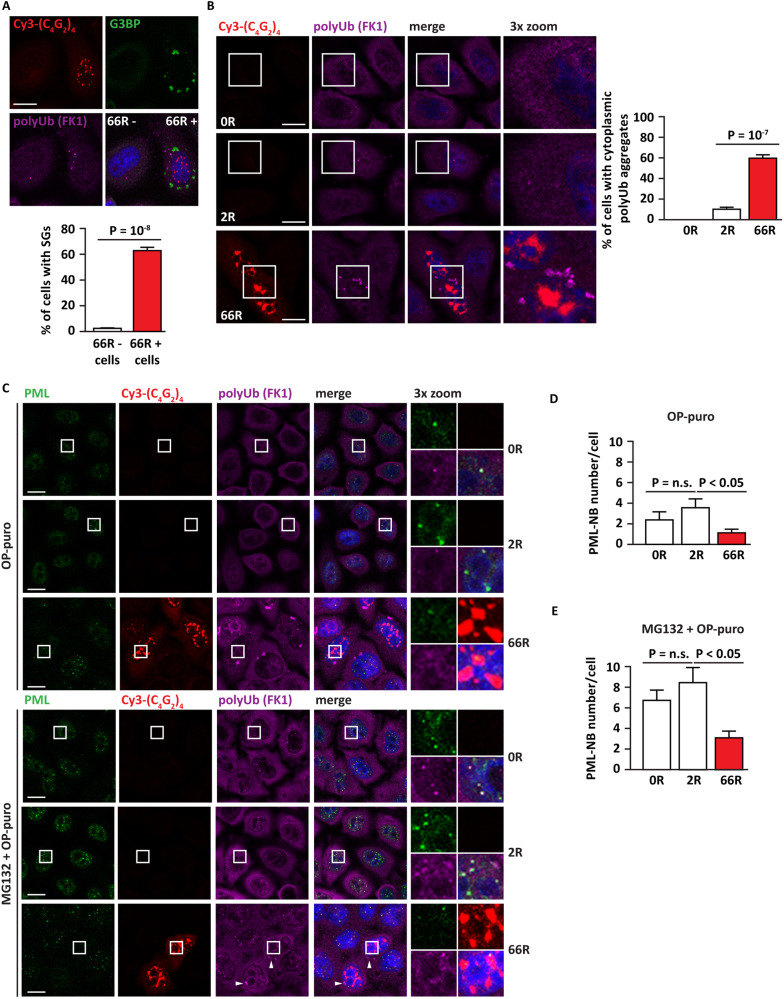
HeLa cells overexpressing expanded C9orf72 repeats display spontaneous SGs and decreased PML-NB numbers. **A** HeLa Kyoto cells were transfected for 24 h with a vector coding for 66 repeats (66 R). Then cells were fixed, subjected to FISH to visualize RNA foci and to immunostaining with antibodies specific for the SG protein G3BP1 and for ubiquitin (clone FK1). Nucleic acid was labeled using DAPI. Scale bar is 10 μm. The percentage of 66 positive (66 +) and 66 negative (66 -) cells with SGs is shown; *n* = 4, −/+ s.e.m. Number of cells quantified: 66 R - (1243); 66 R + (818); Student’s t-test. **B** HeLa Kyoto cells were transfected for 24 h with an empty vector (0 R), or vectors coding for 2 or 66 repeats (2 R, 66 R). Cells were fixed, subjected to FISH to visualize RNA foci and to immunostaining with antibodies specific for polyubiquitin (FK1). Nucleic acid was labeled using DAPI. Scale bar is 10 μm. The quantification of the percentage of cells with cytoplasmic polyubiquitin-positive foci is shown. *n* = 5, −/+ s.e.m. Number of cells quantified: 0 R (430); 2 R (1491); 66 R (762). One-way ANOVA, Post-hoc Bonferroni-Holm test. C-E: HeLa Kyoto cells were transfected for 24 h with an empty vector (0 R), or vectors coding for 2 or 66 repeats (2 R, 66 R). Then cells were either treated with OP-puro (25 μg/ml) alone or with MG132 (20 μM) for 3 h. Cells were fixed, subjected to FISH to visualize RNA foci and to immunostaining with antibodies specific for PML and polyubiquitin (FK1). The white arrowheads show polyubiquitin-positive cytoplasmic aggregates. Nucleic acid was labeled using DAPI. Scale bar is 10 μm. Representative images are shown in (**C**). **D**, **E** The average number of PML-NBs in 0 R, 2 R and 66 R expressing cells shown in (**C**). *n* = 3, −/+ s.e.m. Number of cells quantified in (**D**): 0 R (588); 2 R (567); 66 R (390). Number of cells quantified in (**E**): 0 R (482); 2 R (613); 66 R (442). One-way ANOVA, Post-hoc Bonferroni-Holm test.

The expansion of the GGGGCC (G_4_C_2_) hexanucleotide repeat in the first intron of the C9ORF72 gene is one of the most frequent genetic causes of ALS and FTD [2]. Transcripts containing C9-G_4_C_2_ repeats form nuclear RNA foci that cause cell toxicity with different mechanisms [39–41]. In particular, G_4_C_2_ RNA foci promotes SG formation in a repeat length-dependent manner [42]. Based on these data, we overexpressed in HeLa cells a vector coding for 66 G_4_C_2_ repeats (66R) [43]. Vectors coding for no (0R) or 2 (2R) repeats were employed as controls. In line with previous findings [42], 66R overexpressing cells were characterized by the accumulation of nuclear RNA foci; circa 60 % of the transfected cells were characterized by the formation of SGs (here referred to as spontaneous SGs) (Fig. 6A). In addition, when compared to cells overexpressing 0R or 2R, used as controls, we noticed that the 66R overexpressing cells also showed the accumulation of cytoplasmic aggregates positive for polyubiquitin chains (Fig. 6B), which is a typical hallmark of *C9orf72* ALS cases [44]. Polyubiquitin chains were absent inside SGs that formed spontaneously in the 66R overexpressing cells (Fig. 6A). This is in line with recent ubiquitinome analysis showing that ubiquitination is required for the disassembly of SGs induced by heat shock, but not by oxidative stress conditions that are induced by mutated *C9orf72* [45–47]. Collectively, this cellular model recapitulates the impairment of the cytoplasmic PQC system observed in ALS-FTD cells and represents a good model to further study whether this is paralleled (or preceded) by alterations of the nuclear arm of the PQC.

To address this question, we quantified the number of PML-NBs in cells expressing 0R, 2R or 66R, using a PML specific antibody, followed by PML-NB automatic segmentation, as previously described [12]. Intriguingly, 66R overexpression per se significantly decreased the number of PML-NBs compared to cells expressing 0R or 2R (Fig. 6C, D).

The number of PML-NB raises upon proteotoxic stress such as proteasome inhibition, to efficiently compartmentalize misfolded proteins, including SUMO-primed proteins and DRiPs [12]. We thus verified whether 66R overexpressing cells could efficiently induce the number of PML-NBs upon treatment with the proteasome inhibitor MG132 and OP-puro. The latter was added to induce the accumulation of DRiPs that are known to be compartmentalized at PML-NBs [12]. As expected, control (0R and 2R expressing) cells treated with MG132 and OP-puro showed a strong increase in the number of PML-NBs, which colocalized with polyubiquitin chains (Fig. 6C, E). By contrast, cells overexpressing 66R showed a significantly lower number of PML-NBs also when challenged with proteasome inhibition and OP-puro (Fig. 6C, E). Together with the data presented in Fig. 3 these results further support the idea that a correlation exists between impaired PML-NB assembly, compartmentalization of nuclear polyubiquitinated proteins and the accumulation of cytoplasmic SGs. Whether these events are directly linked is still unclear.

### Decreased PML-NBs correlate with defects in SG disassembly in iPSC-derived motor neurons expressing expanded *C9orf72*

The transient overexpression of 66R represents a severe stress for the cells, which accumulate spontaneous SGs. To rule out whether the decrease in the number of PML-NBs observed in 66R overexpressing cells may be an artifact due to its high expression levels, we used iPSC lines expressing expanded (pathogenic) *C9orf72* (referred to as C9-ALS) and gene-corrected non-expanded *C9orf72* (referred to as GC) [48]. We first characterized PML-NBs using C9-ALS and GC in the undifferentiated state. Undifferentiated C9-ALS and GC iPSCs were either left untreated (control) or exposed to sodium arsenite, to induce both PML-NB and SG assembly. Cells were fixed either immediately after stress or after recovery in drug-free medium. PML-NBs were segmented using ScanR and automatically quantified under all conditions tested. The C9-ALS and GC iPSC lines showed a similar behavior: PML-NB number was similar under resting conditions between C9-ALS and GC iPSC lines and increased upon stress in both lines (Supplementary Fig. 2A, B). The average size of PML-NB was even slightly, but significantly, higher in undifferentiated C9-ALS compared to GC iPSC lines under all conditions tested (Supplementary Fig. 2C). These data suggest that differences in the dynamics of PML-NBs are not present in the undifferentiated C9-ALS and GC iPSC lines.

**Fig. 7:**
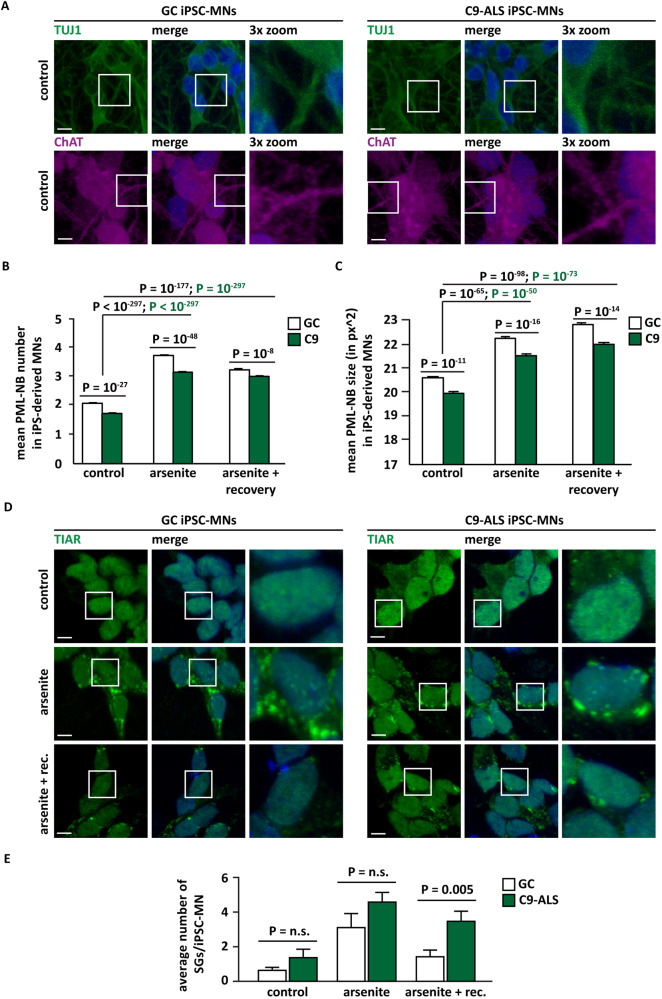
iPSC-MNs expressing expanded G4C2 repeats are characterized by a decreased number of PML-NBs compared to gene corrected iPSC-MNs. **A** C9-ALS and GC iPSCs were differentiated into motor neurons. Cells were fixed and stained with an antibody specific for neuron-specific class III beta-tubulin (TuJ1) and Choline Acetyltransferase (ChAT). Nucleic acid was labeled using DAPI. **B**, **C** C9 and GC iPSCs were differentiated into motor neurons. C9-ALS and GC iPSC-MNs were either left untreated or treated with sodium arsenite (500 μM) for 1 hr; where indicated cells were let to recover in drug-free medium for 2 hrs after treatment ( + rec.). Cells were fixed and stained with an antibody specific for PML. Nucleic acid was labeled using DAPI. PML-NBs were automatically segmented and quantified. The average number of PML-NBs is shown in (**B**). The average size of PML-NBs is shown in (**C**). *N* = 5 independent experiments, +/− s.e.m. Number of cells analyzed/condition: GC, control (8022); C9-ALS, control (6128); GC, arsenite (7188); C9-ALS, arsenite (8691); GC, arsenite + rec. (4997); C9-ALS arsenite + rec. (7829). One-way ANOVA, Bonferroni-Holm test. **D**, **E** GC and C9-ALS iPSC-MNs were either left untreated or treated with sodium arsenite (500 μM) for 1 hr; where indicated cells were let to recovered in drug-free medium for 2 hrs after treatment ( + rec.). Cells were fixed and stained with antibodies specific for the SG markers TIAR or G3BP1. Nucleic acid was labeled using DAPI. Scale bar is 20 μm. Representative images are shown in (**D**). SGs were quantified. The percentage of SGs/cell is shown in (**E**). *N* = 3–4 independent experiments, +/− s.e.m. Number of analysed cells/condition: GC, control (363); C9-ALS, control (340); GC, arsenite (1805); C9-ALS, arsenite (935); GC, arsenite + rec. (912); C9-ALS arsenite + rec. (885). One-way ANOVA, Bonferroni-Holm test.

Then, we differentiated C9-ALS and GC iPSC lines into motor neurons (iPSC-MNs). Differentiation was confirmed by staining with neuron-specific class III beta-tubulin (TUJ1) and Choline Acetyltransferase (ChAT) (Fig. 7A). We next stained PML in differentiated C9-ALS and GC iPSC-MNs, followed by automated quantification. Under all conditions tested, C9-ALS iPSC-MNs were characterized by a significantly lower number of PML-NBs compared to GC iPSC-MNs (Fig. 7B). Upon exposure to arsenite, we observed an increase in the PML-NB number in both C9-ALS and GC iPSC-MNs (Fig. 7B). However, both average PML-NB number and size were significantly lower in C9 compared to GC iPSC-MNs (Fig. 7B, C). The decreased PML-NB number observed in C9-ALS iPSC-MNs upon arsenite treatment correlated with a significant delay in the disassembly of SGs, compared to GC iPSC-MNs (Fig. 7D, E).

PML-NBs are stress-responsive structures and increased transcription of PML contributes to increase PML-NB size and number [49], along with SUMO1 covalent attachment to PML [37]. We thus verified whether, compared to GC iPSC-MNs, C9 iPSC-MNs are characterized by lower expression levels of the key molecules involved in PML-NB assembly: PML, the E2 SUMO conjugating enzyme Ubc9 and SUMO1. By RNAseq analysis, we found that compared to GC iPSC-MNs, C9-ALS iPSC-MNs are characterized by slightly lower levels of PML mRNA, slightly higher levels of Ubc9 and similar levels of SUMO1. Whether these slight differences in the mRNA levels of PML are sufficient to cause a decrease in the number of PML-NBs is unclear (Fig. S2D). Of note, published RNA-Seq analyses performed by independent groups using *C9orf72* mutant iPSC-derived MNs did not identify significant changes in the expression levels of PML, Ubc9 and SUMO1 compared to control cells [50, 51]. In particular, the multi-omic study conducted by The NeuroLINCS Consortium included MNs derived from ten control iPSC lines and ten C9-ALS lines, all showing relevant *C9orf72* pathology [50].

Finally, to further corroborate the results obtained in C9 iPSC-MNs, we employed cortical neurons derived by control and C9-ALS iPSC. Control neurons showed a significantly higher number of PML-NBs compared to C9-ALS neurons (Fig. S3A, B).

We conclude that the expression of expanded *C9orf72* G_4_C_2_ repeats in HeLa cells and iPSC-MNs phenocopies the defects observed upon PML and Ubc9 depletion. These results establish a strong link between SG pathology and PML and identify defective PML-NB assembly and dynamics in C9-ALS cell models.

## Discussion

In this study, we provide evidence for a link between two stress-induced condensates: cytoplasmic SGs and PML-NBs. We show that alterations at the level of PML-NBs correlate with defective SG dynamics in cellular models of ALS-FTD. In addition, we report that the number of PML-NBs is decreased in familial *C9orf72* and FUS ALS-FTD cases. Our results suggest that defects at the nuclear arm of the PQC, whose key players are the PML-NBs, promote SG aggregation and pose a threat for the cytoplasmic arm of the PQC.

This interpretation is based on our finding that, upon depletion of PML or Ubc9, DRiPs fail to be compartmentalized in the nucleus and accumulate inside SGs, delaying their disassembly. Our data integrate previous findings showing that inhibition of SUMOylation and depletion of PML or RNF4 impairs the nuclear quality control of mutated ALS-linked FUS and causes its accumulation inside SGs, delaying their disassembly [20]. Importantly, we extend the link between defective PML-NB assembly and defective SG dynamics to *C9orf72*-cellular models, including iPSC-MNs. Thus, failure to compartmentalize aggregation-prone proteins, including DRiPs and ALS-linked RBPs in the nucleus poses a threat for the restoration of protein homeostasis in the cytoplasm, impairing SG dynamics. To what extent the SGs accumulating as a consequence of defective PML-mediated nuclear quality control contribute to the formation of the cytoplasmic pathological aggregates in ALS-FTD cellular models is still unclear.

Next, we demonstrate that the number of PML-NBs were strongly reduced in the cortical and hippocampal neurons, as well as in the lumbar spinal cord α- MNs of both *C9orf72* and FUS ALS-FTD patients. So far, PML-NBs were implicated in polyglutamine diseases, where PML often forms a ring-like structure surrounding nuclear inclusions [16]. Using cellular and animal models it was shown that PML-NB induction improves the degradation of expanded ataxin-1 and ataxin-7, linked to spinocerebellar ataxia type 1 and 7 [13, 52, 53]. Conversely, disruption of PML-NBs increased the accumulation of nuclear ataxin-1 and 7 aggregates [13, 53], supporting the idea that loss of PML function contributes to protein aggregation and toxicity in polyglutamine diseases. Although ALS-FTD are mainly characterized by the accumulation of cytoplasmic inclusions positive for phosphorylated TDP-43, ubiquitin, and p62 [5], nuclear aggregates have been also documented. Nuclear aggregates that colocalize with PML, ubiquitin, and proteasome have been identified in hippocampal pyramidal neurons of ALS patients [54, 55]. Intranuclear inclusions positive for PML, SUMO1, but also p62 and VCP were also described in a subset of patients with FTD [16, 56]. Finally, neuronal intranuclear inclusions positive for SQSTM1/p26, but negative for phosphorylated TDP-43, have been found in hippocampal and cerebellar neurons in C9orf72 ALS-FTD patients [57]. We did not observe nuclear protein aggregates containing phosphorylated TDP-43, mutated FUS or dipeptide repeat proteins (polyGA) in the ALS-FTD samples analyzed. Instead, we found a general decrease in the number of PML-NBs in the brain and spinal cord of familial *C9orf72* and FUS ALS-FTD. Rather than co-aggregation with PML, as shown in polyglutamine diseases, our data suggest a more complex mechanism in ALS-FTD, where an alteration of the interplay between the nuclear and cytoplasmic arms of the PQC would indirectly contribute to cytoplasmic protein aggregation. Our data reveal reduced PML-NB number as a common feature in familial *C9orf72* and FUS ALS-FTD patients, although we still do not understand what causes this decrease and how mechanistically it contributes to disease pathogenesis/progression.

Amongst the factors that regulate PML-NB assembly are PML transcriptional upregulation and PML conjugation to SUMO1 [37, 49]. Whether the reduced PML-NB number observed in the cellular models and ALS-FTD autopsy samples analyzed here is the result of lower PML transcriptional regulation is still unclear. Yet, current data from independent groups analyzing MNs derived from several control and *C9orf72* iPSC lines, as well as *C9orf72* ALS postmortem cervical spine tissues [50, 51], showed no significant differences in the PML mRNA levels. In addition, compared to control subjects the expression levels of PML, Ubc9, and SUMO1 were also not significantly different in the frontal cortex tissue from a cohort of patients affected by Frontotemporal lobar degeneration (FTLD), regardless of the *C9orf72* repeat expansion [58]. Globally, these observations suggest that the mild decrease of PML transcription observed in the C9-ALS iPSC-MNs may not be at the basis of the reduced PML-NB number observed.

Concerning the question how mechanistically a decrease in the number of PML-NBs would contribute to disease pathogenesis/progression, our data suggest that dipeptide repeat proteins generated from mutant *C9orf72* and endogenous DRiPs are handled by the same proteostasis machinery. Although these proteins are normally rapidly degraded by the proteasome, they may escape proteasomal degradation, especially with aging, when the proteostasis capacity declines, thereby accumulating at the level of nuclear hubs such as PML-NBs and nucleoli [4, 12]. In addition, proline-arginine-rich dipeptide repeat proteins physically associates with proteasomes and inhibit the degradation of ubiquitylated substrates [59]. Thus, the concomitant production of DRiPs and dipeptide repeat proteins may “poison” the nuclear proteostasis machinery [12, 60]. Failure of handling nuclear DRiPs, as a consequence of reduced PML-NB numbers and impaired proteasomes, could contribute to irreversible protein aggregation in the cytoplasm and at the level of SGs in ALS-FTD, while also impairing nuclear proteostasis.

Despite its role in nuclear quality control, PML also participates to the maintenance of genome integrity. We cannot exclude that PML-NB impairments in ALS-FTD patient cells may affect PML functionality in DNA damage sensing and repair. Of note, DNA damage is emerging as an important pathomechanism in ALS-FTD [61, 62]. The ALS-linked proteins TDP-43 and FUS, but also p62, VCP, and senataxin all play a role in the maintenance of genome integrity [62]. Curiously, these proteins can also colocalize with PML-NBs. For example, p62 accumulates with polyubiquitylated proteins at PML-NBs when nuclear export is impaired and forms proteolytically active nuclear condensates that facilitate protein clearance [63, 64]. Instead, upon proteotoxic stress conditions VCP is recruited at PML-NBs along with DRiPs to orchestrate their clearance [12]. Thus, the functions of PML-NBs and co-players such as p62 and VCP, whose mutations have been also linked to ALS-FTD [65–67], at the level of nuclear proteostasis and genome integrity seem to be interconnected. It has been proposed that aggregating proteins can sequester factors required for DNA repair and can limit the availability of free ubiquitin molecules, impairing the ubiquitin-dependent signaling during the DNA damage response [12, 68]. In addition, genomic instability and decreased DNA damage sensing due to PML-NB dysfunction may introduce de novo mutations, generating mutated proteins and DRiPs, which can further challenge proteostasis [68]. Future studies will need to clarify how loss of proteostasis and genomic instability are interconnected and how the decreased number of PML-NBs may contribute to both protein aggregation and DNA damage in ALS-FTD. Intriguingly, the number of PML-NBs decreases with aging and inversely correlates with the number of γH2AX foci, further supporting the intimate link between these two fundamental processes [69].

Finally, recent findings show the existence of a link between PML and autophagy. The autophagy adapter protein p62/SQSTM1 sequesters ubiquitinated proteins at PML-NBs [63], and the PML protein has been localized to lysosomes [70]; in addition, a fraction of cytoplasmic PML localized at mitochondria-associated membranes (MAMs) inhibits autophagosome formation, repressing autophagy [71]. Together these data suggest the existence of complex mechanisms regulating the cytoplasmic and nuclear PQC, with critical players working in both compartments. Future studies will need to address whether cytosolic PML may also indirectly affect SG dynamics and whether loss of cytoplasmic PML could participate in ALS-FTD progression.

In summary, our data identify a reduction in the number of PML-NBs in different cellular models, including iPSC-MNs expressing mutated *C9orf72* linked to ALS-FTD and in the brain and spinal cord from patients affected by ALS-FTD. Defective PML function and impaired nuclear PQC may contribute to protein aggregation and SG dysfunction in the cytoplasm. Thus, approaches aimed at restoring PML functionality may help maintaining proteostasis and genome stability, delaying disease progression.

## Materials and methods

### Cell lines

HeLa Kyoto cells were cultured in high glucose DMEM supplemented with 10% fetal bovine serum, penicillin and streptomycin antibiotics, at 37 °C in a 5% CO_2_ incubator. Hela Kyoto cells stably expressing GFP-G3BP2 and GFP-PML, as well as the vector coding for mCherry-PML, were a kind gift from Dr. A.A. Hyman and Dr. S. Alberti; these cell lines were cultured in the presence of geneticin (Life Technologies, 400 μg/ml) [29].

The induced pluripotent stem cells (iPSCs) used in this study were previously generated and characterized [48]. Undifferentiated iPSCs were grown in mTeSR medium (Stemcell Technologies). Differentiation into motor neurons (MNs) was obtained by cultivating the cells in maturation medium consisting of N2B27 [DMEM/F-12 and Neurobasal medium (1:1) supplemented with PS/G, N2, and B27 supplement (N2B27, Thermo Fisher)], supplemented with 200 mM AA, 0.1 mM dibutyryl cyclic-AMP sodium salt (Sigma-Aldrich), 1.5 mg/ml transforming growth factor beta 3 (Peprotech), 4 mg/ml GD, 2 mg/ml BD, and 5 mg/ml activin A (aA, eBioscience), as previously described [48].

Cortical neurons were derived from control and C9 lines (expressing expanded, pathogenic, C9orf72, referred to as C9-ALS) following a previously published protocol [72]. Briefly iPSCs were regularly cultured in E8 media. To achieve cortical neuron differentiation, iPSCs were splitted with accutase and replated in induction media (F12, 1% N2). After 3 days in induction media cells were replated on PLO (poly-ornithine) in maturation media (brainphys, 1% N2, 2% B27, NT3 10 nM, BDNF 10 nM).

### Transfection, protein extraction, and immunoblotting

Cells were transfected with siRNAs and cDNAs using Lipofectamine 3000 (Life Technologies) following manufacturer instructions. 24 and 72 h after transfection of cDNAs or siRNA, respectively, cells were processed for immunoblotting, RT-qPCR.

For immunoblotting, total protein extracts were prepared by lysing the cells in Laemmli sample buffer, followed by sonication. Protein samples were boiled for 3 min at 100 °C and reduced with β-mercaptoethanol, followed by SDS-PAGE. Proteins were transferred onto nitrocellulose membranes and analyzed by western blotting.

### RNA extraction, RT-qPCR, and transcriptomic

For RT-qPCR, total RNA was isolated using Trizol reagent (R2050-1-200, Zymo research), followed by purification with RNA clean & concentrator Zymo kit (25-R1017, Zymo research) according to the manufacturer’s instructions. Reverse transcription was performed using 0.25 μg of RNA and Maxima First-strand cDNA Synthesis Kit with dsDNase (K1672, Thermo Fisher), according to the manufacturer’s instructions. PCR amplification was performed using Maxima SYBR Green qPCR Master Mix polymerase (Thermo Fisher). The relative changes in the levels of human PML, Ubc9, and SUMO1 were analyzed using CFX96 Touch Thermal cycler (Bio-Rad, Hercules, CA, USA). The primers used were all purchased from Eurofins-Genomics and are listed below: PML for (CCGCAAGACCAACAACATCTT); PML rev (CAGCGGCTTGGAACATCCT); Ubc9 for (GGACTTTGAACATGTCGG); Ubc9 rev (CCGAAGGGTACACATTCGG); SUMO1 for (CCTCAGTTGAAGGTTTTGCC); SUMO1 rev (GGAGCGAGGTTCTGCTTACC); RPL0 for (TTAAACCCTGCGTGGCAATCC); RPL0 rev (CCACATTCCCCCGGATATGA). qPCR was performed as follows: one cycle of denaturation (95 °C for 3 minutes), 40 cycles of amplification (95 °C for 10 seconds, 60 °C for 30 seconds). Data were analyzed with Bio-rad CFX Manager 3.1 (Windows 7.0).

RNAseq analysis on C9 and GC iPSC-MNs was described previously [48] and the resulting data deposited in GEO as accession numbers GSE143743 and GSE143744.

### Fluorescence in situ hybridization (FISH)

HeLa cells were seeded on polylysine-coated glass coverslip and transfected using Lipofectamine 2000 (Life Technologies) with either an empty vector (0 R), or vectors coding for 2 and 66 G_4_C_2_ repeats (2 R and 66 R), kindly provided by Prof. Leonard Petrucelli [43]. Cells were washed in PBS pH 7.4, fixed with 4% formaldehyde in PBS pH 7.4 for 10 min at room temperature, washed twice in 70% ethanol and then rehydrated in PBS 5 mM MgCl_2_ for 30 min at room temperature. Pre-hybridization was performed by incubating the cells with 35% formamide in SSC buffer supplemented with 10 mM Na Phosphate, pH 7.0 for 30 min. Hybridization was performed by incubating the cells overnight at 37 °C in a humidified chamber with 5 ng of Cy3-(C_4_G_2_)_4_ (Sigma-Aldrich), 30% formamide, 10 mM sodium phosphate, pH 7.0, 10 µg of E. coli tRNA, 10 µg of sonicated salmon sperm DNA, 1x hybridization buffer (10% dextrane sulfate, 2x SSC, 0.2% BSA). The day after, cells were washed twice for 30 min at 37 °C in 35% formamide 2X SSC, 10 mM Na Phosphate pH 7.0, followed by additional washes at room temperature in 0.2X SSC, 0.1% Triton-X100. Cells were then subjected to immunostaining using specific primary antibodies, followed by incubation with fluorescently labeled secondary antibodies and DAPI (as described below).

### Immunofluorescence on cultured cells and labeling of nascent peptides with OP-puro

HeLa cells were grown on polylysine-coated glass coverslip. Undifferentiated iPSCs were grown on matrigel-coated 96-well plates with clear bottoms (Greiner Bio-ONe, 655090), while iPSC-MNs were grown on biolaminin-coated 96-well plates with clear bottoms (Greiner Bio-ONe, 655090). After washing with cold PBS, cells were fixed with 3.7% formaldehyde in PBS for 9 minutes at room temperature, followed by permeabilization with ice-cold acetone for 5 minutes at −20 °C. Alternatively, cells were fixed with ice-cold methanol for 10 minutes at −20 °C. PBS containing 3% BSA and 0.1% Triton X-100 was used for blocking and incubation with primary and secondary antibodies.

Labeling of newly synthesized proteins was performed by incubating HeLa cells with 25 μM O-Propargyl-puromycin (OP-puro, NU‐931‐05 Jena Bioscience) for the indicated time-points, alone or in presence of the proteasome inhibitor MG132 (20 μM). OP-puro labeled peptides were detected by click chemistry as previously described [73]. Samples were then subjected to immunostaining with specific antibodies to label PML, polyubiquitinated proteins, SUMO1 or SUMO2/3.

### Immunofluorescence on iPSC-derived motor neurons and iPSC-derived cortical neurons

GC and C9 iPSC-derived motor neurons (iPSC-MNs) were treated with 500 µM sodium arsenite for 1 hr to induce SGs. For the “recovery” samples, arsenite-containing medium was removed and replaced with fresh medium for 2 h. iPSC-MNs were fixed using 4% PFA for 20 minutes, followed by immunostaining with neuron-specific class III beta-tubulin/TuJ1, Choline Acetyltransferase/ChAT, TIAR or G3BP1 (not shown) specific antibodies. Nucleic acid was labeled with DAPI. Confocal microscopy at 63x magnification was used to acquire images, and quantification was carried out using ScanR software (Olympus).

iPSC-derived cortical neurons after 25 days in cultured were fixed for 20 minutes in 4% PFA, washed with PBS and permeabilized with PBS, 1% BSA, 10% FBS and 0.05% Triton X-100. Primary antibodies for PML, Map2, and G3BP1 were incubated o/n at 4° in 0.1% BSA. Secondary antibodies were incubated for 1 hour at RT in 0.1% BSA. DAPI was diluted 1:3000 and incubated for 10 minutes. Images were acquired with Nikon A1-R confocal microscope with a 60X objective.

### High-content imaging assay with ScanR Analysis software (Olympus), quantification, and statistical analyses

SGs and PML-NB were quantified in fixed HeLa cells, GFP-PML HeLa Kyoto cells and human iPSCs. The data were imported into the ScanR (Olympus) software. PML-NBs were segmented based on PML signal using edge detection algorithm, except for data shown in Fig. 5B where the SUMO1 signal was used as a proxy for PML-NB segmentation. Nuclei were segmented based on the DAPI signal intensity. PML-NB number/nucleus and PML-NB area was automatically calculated. Concerning DRiP enrichment inside SGs, first, SGs were segmented based on TIA-1 signal using edge detection algorithm. Then, the mean fluorescence intensity of DRiPs was measured in each detected SG and in an area surrounding the SG. The relative enrichment of DRiPs in individual SGs was calculated as a ratio of mean fluorescence intensity inside the SG divided by mean intensity in the region surrounding the SG. The values were plotted as column graphs representing the fraction of SGs with enrichment > 1.5.

Statistical analyses were performed using Student’s t-test for comparisons between two groups or One-way ANOVA, followed by Bonferroni-Holm post-hoc test for comparisons between three or more groups using Daniel’s XL Toolbox or GraphPad Prism6 software.

### Chemicals, primary and secondary antibodies

Chemicals: sodium arsenite (Carlo Erba); Z‐Leu‐Leu‐Leu‐al (MG132; C2211, Sigma‐Aldrich); Cycloheximide (C7698, Sigma‐Aldrich); ML-792 (HY-108702, Medchemexpress); DAPI (SC3598, Santa Cruz Biotechnology).

Primary antibodies: PML (ab179466 and ab53773, abcam 1:100); TIAR (610352, BD Biosciences, 1:100); G3BP1 (611126, BD Biosciences, 1:100); neuron-specific class III beta-tubulin/TuJ1 (60052, Stemcell Technologies, 1:500); Choline Acetyltransferase/ChAT (SAB2500236, Merck, 1:75); polyUb proteins (FK1; BML‐PW8805‐0500, Enzo, 1:100); TUBA4A (T6074, Merck, 1:1000); SUMO1 (ab49767 and ab219724, abcam, 1:100); SUMO2/3 (ab3742, abcam, 1:100 and 10947-1-AP, proteintech 1:100).

Secondary antibodies: mouse IgG HRP linked whole ab (NXA931, GE Healthcare, 1:5000); rabbit IgG HRP linked whole ab (NA934, GE Healthcare, 1:5000); Alexa Fluor™ 594 Azide (A‐10270, Thermo Scientific, 10 μM); Donkey anti‐Mouse IgG (H + L), Alexa Fluor® 594 (A‐21203, Thermo Scientific, 1:1000); Donkey anti‐Mouse IgG (H + L), Alexa Fluor® 488 (A‐21202, Thermo Scientific, 1:1000); Donkey anti‐Rabbit IgG (H + L), Alexa Fluor® 594 (A‐21207, Thermo Scientific, 1:1000); Donkey anti‐Rabbit IgG (H + L), Alexa Fluor® 488 (A‐21206, Thermo Scientific, 1:1000).

### Human post-mortem tissue

The post-mortem tissue were obtained within 6–30 h after death (Supplementary Table 1). Human post-mortem brain and spinal cord samples fixed in buffered formalin (*n* = 4 fALS-*FUS* (R521C) patients, *n* = 5 *C9orf72*-fALS patients and *n* = 4 age-matched controls) were obtained from the archives of the Department of Neuropathology, Amsterdam UMC, University of Amsterdam. All patients fulfilled the diagnostic criteria for ALS (El Escorial criteria) [74] as reviewed independently by two neuropathologists. The controls included in the present study were adult individuals without any history of neurological disease, based on their last clinical evaluation. The demographic details of all the fALS patients together with the normal non neurological controls included in this study are summarized in Supplementary Table 1.

### Diaminobenzidine (DAB) immunohistochemistry

Paraffin sections (5 µm) were mounted on poly-L-lysine coated slides and placed to dry (37 °C overnight) and then processed for immunohistochemistry described in detail elsewhere [75, 76]. At first, the sections were deparaffinized in xylene for 20 min and then rehydrated in 100%, 96%, and 70% ethanol for 5 min each followed by endogenous peroxidase quenching (0.3% H_2_O_2_ in methanol) for 20 min. Antigen retrieval was performed in these sections by heating them in citrate buffer, pH 6 (DAKO), for 20 min in a pressure cooker. After washing in PBS, sections were incubated with primary PML antibody (EPR16792, ab179466, 1:100) and C90RF72/C9RANT poly-GA antibody (MABN889, Millipore, 1:100), FUS antibody (ab84078, Abcam, 1:100) or phosphorylated TDP-43 (pTDP-43) antibody (TIP-PTD-M01, Cosmo Bio Co.LTD, 1:100) for 1 h at room temperature or 4 °C overnight. After washing in PBS, sections were incubated with appropriateHRP-linker secondary antibody (ImmunoLogic, Duiven, The Netherlands) for 30 min at room temperature. DAB reagent (ImmunoLogic, ready to use) was used to visualize antibody binding. The sections were then counter-stained with 6% hematoxylin for 3 min. All procedures were performed at room temperature.

### Immunofluorescence labeling of human post-mortem tissue

Double immunofluorescence labeling was performed as described previously [75, 76]. In brief, deparaffinised tissue sections were boiled in citrate buffer, pH 6 (Dako), for 20 min in a pressure cooker for antigen retrieval. Sections were blocked with 10% normal goat serum (Life Technologies), for 1 h at room temperature, and incubated with the required primary antibody (FUS^p-Y526^ and FUS, pAbl; dilutions 1:100 for each antibody), at 4 °C overnight. After washing in TBST for 10 min the sections were incubated with Alexa conjugated secondary antibody (dilution, 1: 500 in PBS) at room temperature for 2 h. Sections were washed in TBST (2× 10 min) and stained for 10 min with 0.1% Sudan Black in 80% ethanol to suppress endogenous lipofuscin auto-fluorescence and then washed for 5 min in TBST and mounted with Vectashield mounting medium (Vector Laboratories) containing DAPI. Images were obtained with a laser scanning confocal microscope (LSM 700; Zeiss) using 40× and 63X objectives (Zeiss). Images were acquired by averaging 4 scans per area of interest resulting in an image size of 1024 × 1024 pixels. For quantification only low-resolution images were used. The laser intensity was kept constant for all of the sections examined. Images were analysed using ZEN (Blue edition) 2009 and IMAGE J software.

## Supplementary information

Supplementary data revised

CDDis checklist

Original Data File

## Data Availability

All data generated or analysed during this study are included in this published article (and its supplementary information files).
