# Supplementary material for: Loss of PML nuclear bodies in familial amyotrophic lateral sclerosis-frontotemporal dementia
Source: Cell Death Discov. 2023 Jul 15;9:248. doi: 10.1038/s41420-023-01547-2 (PMC10349866; doi:10.1038/s41420-023-01547-2)
Supplement: Supplementary file 1 — Supplementary data revised [file 41420_2023_1547_MOESM1_ESM.pdf]

Loss of PML nuclear bodies in familial Amyotrophic Lateral Sclerosis-Frontotemporal Dementia

Francesco Antoniani[1,#], Marco Cimino [1,#], Laura Mediani[1,*], Jonathan Vinet[2], Enza M. Verde[1], Alfred Yamoah[3], Priyanka Tripathi[3], Eleonora Aronica[4], Maria E. Cicardi[5], Davide Trotti[5], Jared Sterneckert[6], Anand Goswami[3,7,8,†*], Serena Carra[1,†*]

Supplementary Table 1. Patients examined in this study. PMI = Post mortem interval.

**Case No.**	**Age**	**Gender**	**Cause of death**	**PMI (hrs)**	**Clinical diagnosis**	**Pathological diagnosis**	**pTDP-43**
1	71	F	Sepsis	18	-	***Normal***	-
2	70	M	Heart failure	7	-	***Normal***	-
3	81	M	Organ failure	16	-	***Normal***	-
4	54	M	Sepsis	15	-	***Normal***	-
1	61	M	unknown	24	fALS-FTD	***C9orf72***	+
2	51	M	pneumonia	12	fALS-FTD	***C9orf72***	+
3	64	F	respiratory	24	fALS-FTD	***C9orf72***	+
4	53	M	respiratory	36	fALS-FTD	***C9orf72***	+
5	68	M	euthanasia	12	fALS-FTD	***C9orf72***	+
1	40	M	unknown	12	fALS-FTD	***FUS***	-
2	35	F	pneumonia	12	fALS-FTD	***FUS***	-
3	70	F	respiratory	12	fALS-FTD	***FUS***	-
4	40	F	respiratory	26	fALS-FTD	***FUS***	-

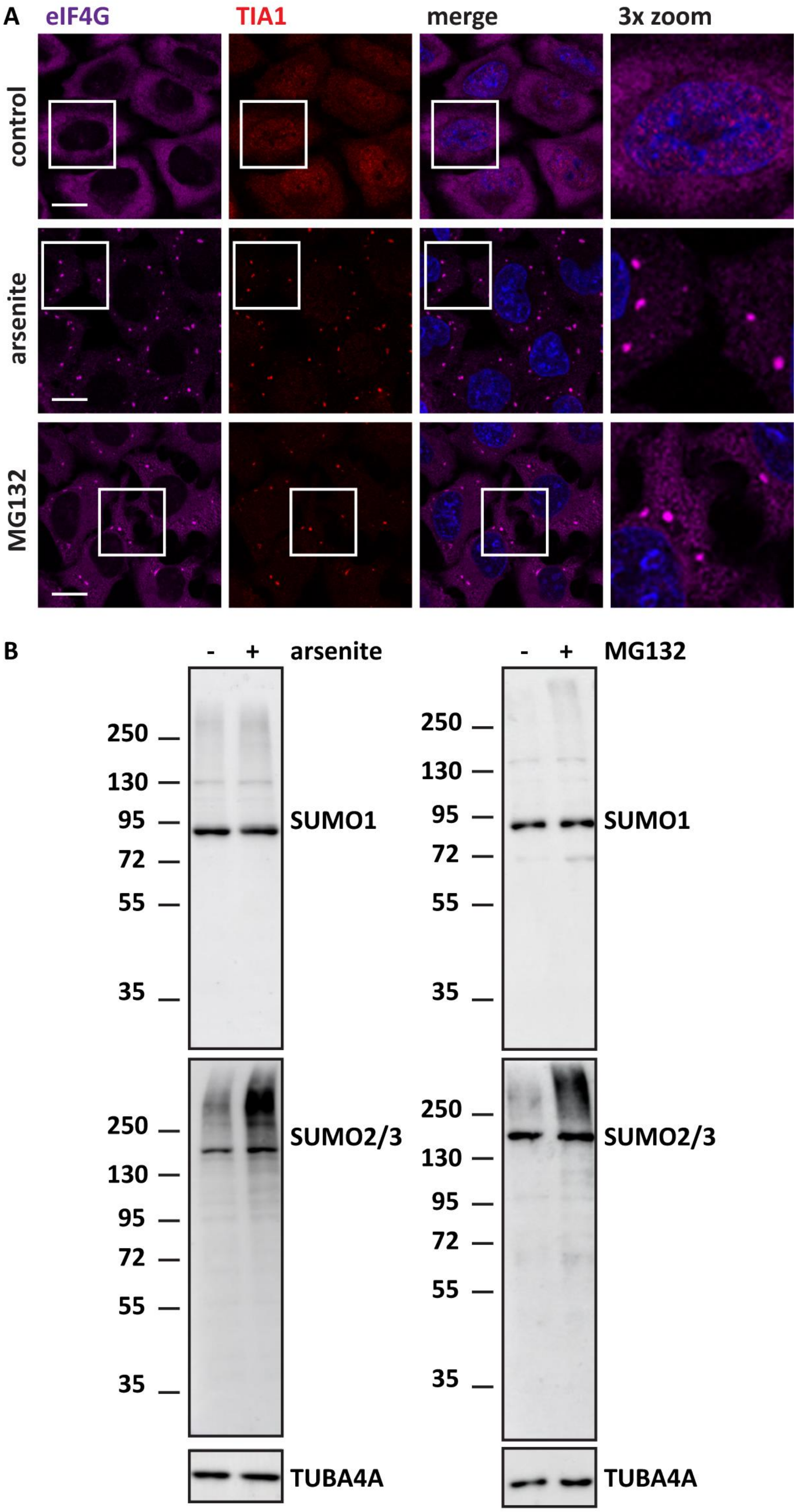
A
eIF4G
TIA1
merge
3x zoom
control
arsenite
MG132
B
- + arsenite
- + MG132
250
130
95
72
55
35
SUMO1
SUMO2/3
TUBA4A

Supplementary Fig. 1. Proteotoxic stress conditions that elicit SG formation induce protein SUMOylation.

A: HeLa Kyoto cells were either left untreated or exposed to sodium arsenite (500 µM) for 45 min or MG132 (20 µM) for 3 hrs. Cells were fixed and immunostained with antibodies specific for the SG markers eIF4G and TIA1. Nucleic acid was labelled using DAPI. Scale bar is 10 µm.

B: HeLa Kyoto cells were treated as described above and protein extracts were subjected to SDS-PAGE followed by immunoblotting with antibodies specific for SUMO1 and SUMO2/3. TUBA4A was used as loading control.

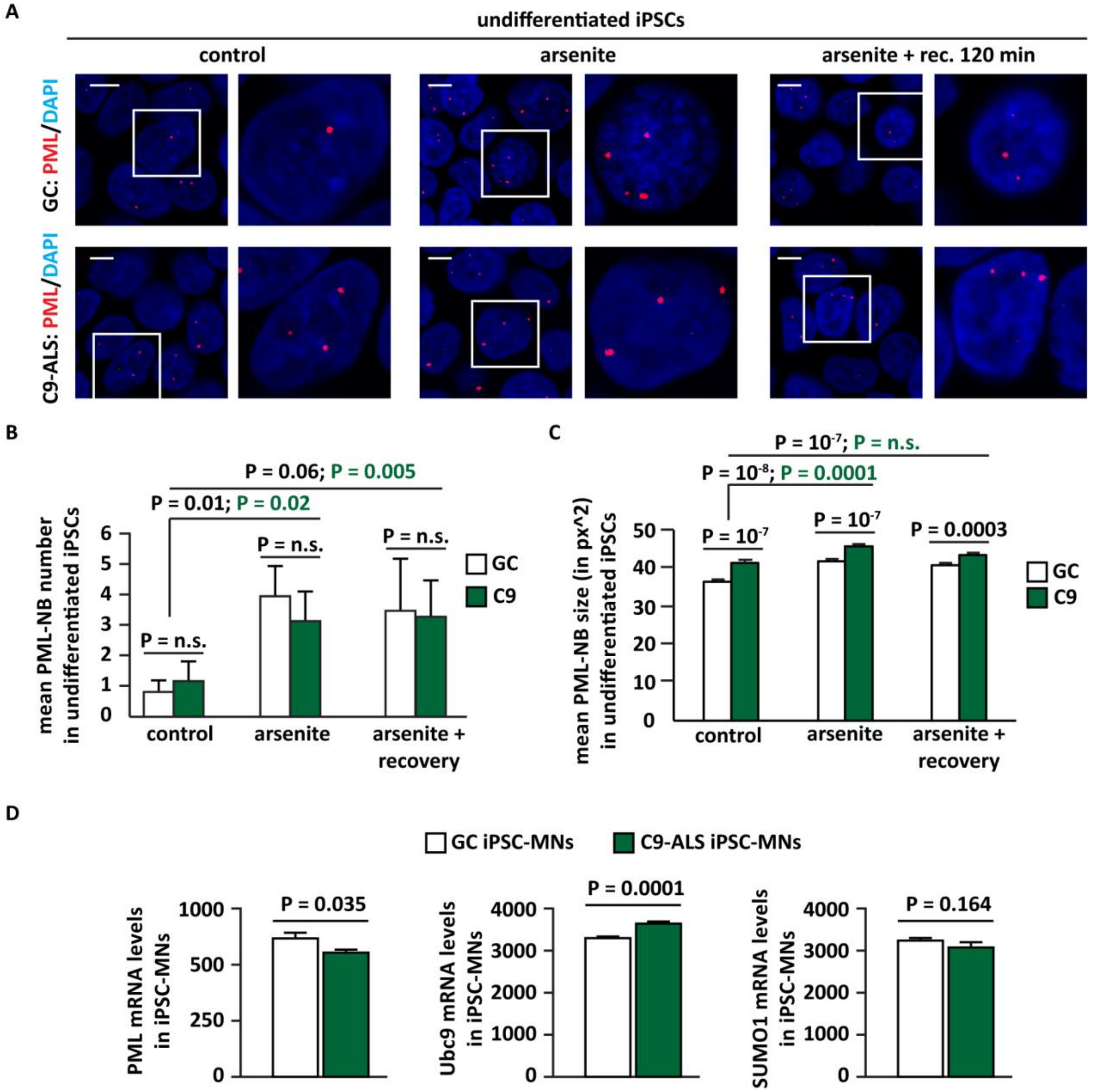

Supplementary Fig. 2: Undifferentiated iPSCs expressing expanded C9orf72 repeats show similar numbers of PML-NBs compared to control iPSCs.

A-C: C9 and GC iPSCs were grown under non differentiating conditions. Cells were either left untreated or treated with sodium arsenite (500 µM) for 1 hr; where indicated cells were let to recover in drug-free medium for 2 hrs after treatment (+ rec.). Cells were fixed and stained with an antibody specific for PML. Nucleic acid was labelled using DAPI. Scale bar is 20 µm. PML-NBs were automatically segmented and quantified. The average number of PML-NBs is shown in B.

The average size of PML-NBs is shown in panel C. N = 3 independent experiments, +/- s.e.m. Number of cells analyzed/condition: GC, control (1190); C9, control (1078); GC, arsenite (842); C9, arsenite (974); GC, arsenite + rec. (784); C9 arsenite + rec. (920). One-way ANOVA, Bonferroni-Holm test.

D: Total RNA was extracted from GC and C9-ALS iPSC-MNs and the expression levels of PML, Ubc9 and SUMO1 was analyzed by RNAseq in three independent samples, +/- s.e.m; Student's t-test.

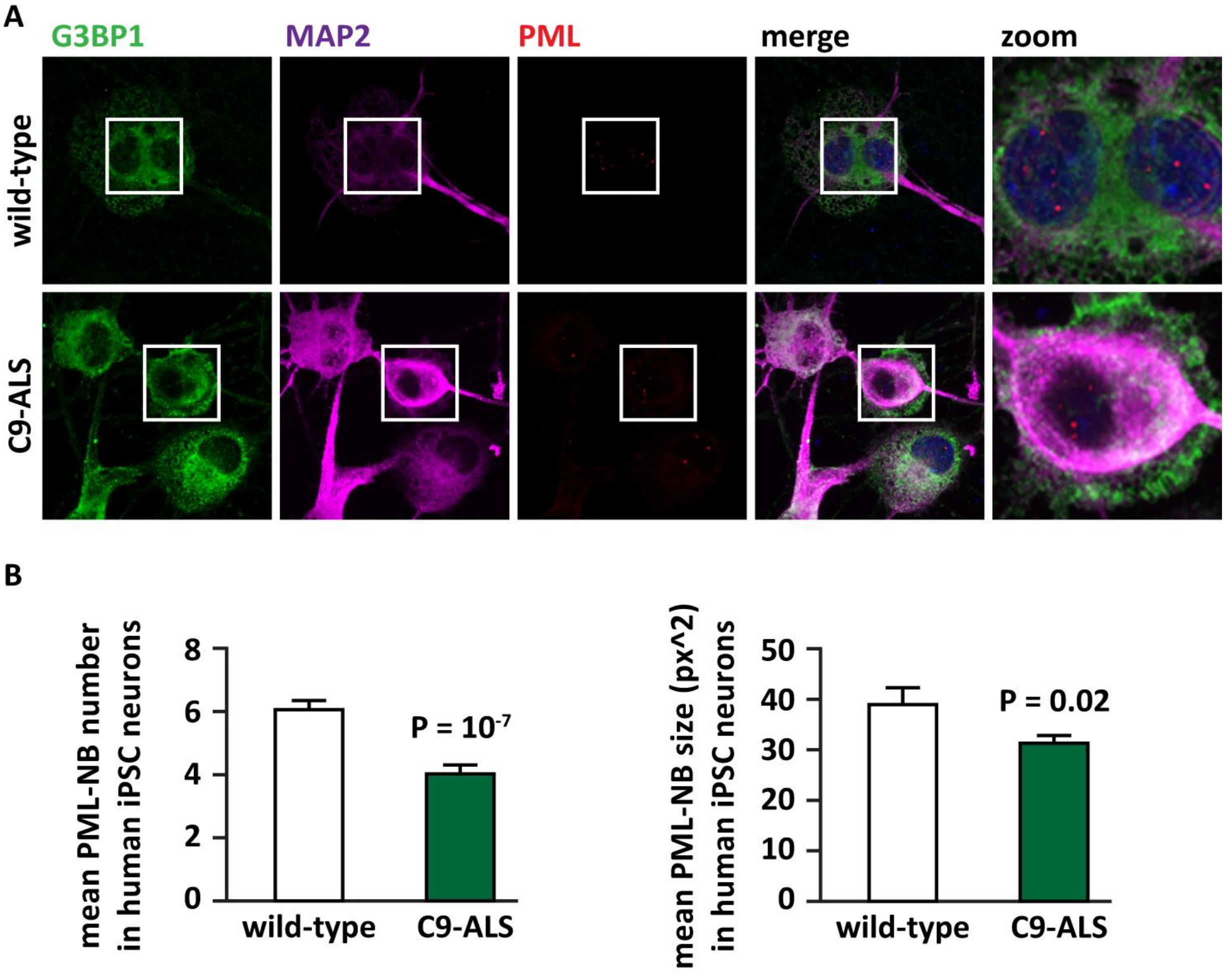

Supplementary Fig. 3: Human iPSC-derived neurons expressing expanded C9orf72 show a decreased number of PML-NBs compared to control neurons.

A. Confocal microscopy of human iPSC-derived neurons wild-type or expressing expanded C9orf72 showing staining of G3BP1 (1:500, Proteintech, 13057-2-AP), MAP2 (1:2000, Novus Biologicals, NB300-213) and PML (1:100, Santa Cruz Biotechnology, sc-377390), followed by incubation with the secondary antibodies Alexa Flour 546 Goat anti-mouse (1:1000, Invitrogen, A11030), Alexa Flour 647 Goat anti-chicken (1:1000, Invitrogen, A32933) and Alexa Flour 488 Goat anti-rabbit (1:1000, Invitrogen, A11008). Nucleic acid was labelled with DAPI. B. PML-NBs were automatically segmented and quantified. The average number and the average size of PML-NBs is shown. Number of neurons analyzed: 84 (wild-type); 70 (C9-ALS).
