# Supplementary figures and images for: Loss of PML nuclear bodies in familial amyotrophic lateral sclerosis-frontotemporal dementia
Source: Cell Death Discov. 2023 Jul 15;9:248. doi: 10.1038/s41420-023-01547-2 (PMC10349866; doi:10.1038/s41420-023-01547-2)

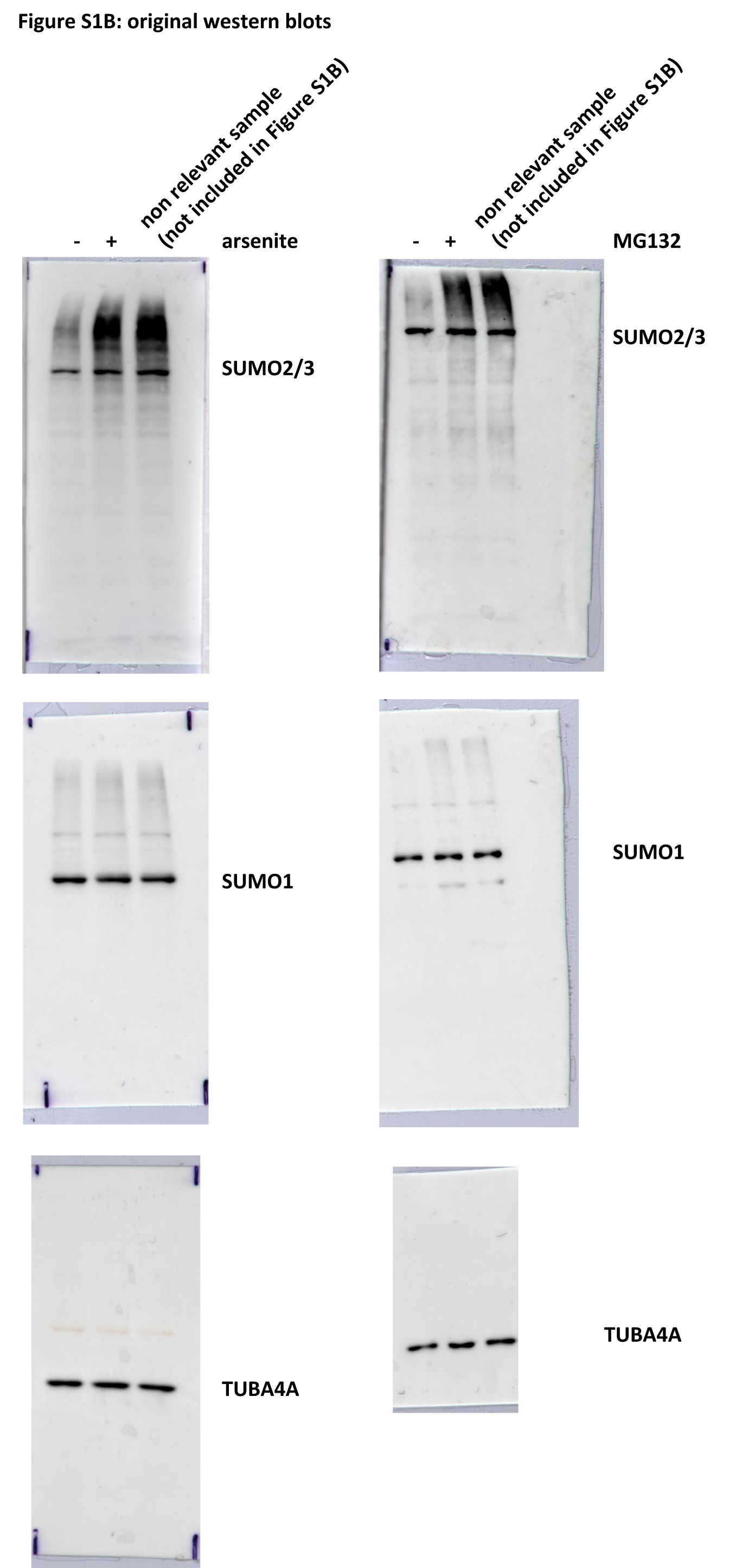

Supplement: Supplementary file 3 — Original Data File [file 41420_2023_1547_MOESM3_ESM.jpg]
